# A Review of Classical Swine Fever Virus and Routes of Introduction into the United States and the Potential for Virus Establishment

**DOI:** 10.3389/fvets.2018.00031

**Published:** 2018-03-05

**Authors:** Vienna R. Brown, Sarah N. Bevins

**Affiliations:** ^1^Oak Ridge Institute for Science and Education (ORISE), Oak Ridge, TN, United States; ^2^United States Department of Agriculture, Animal and Plant Health Inspection Service, Wildlife Services, National Wildlife Research Center, Fort Collins, CO, United States

**Keywords:** classical swine fever, viral introduction, domestic swine, feral swine, emergency preparedness

## Abstract

Classical swine fever (CSF) is caused by CSF virus (CSFV) which can be the source of substantial morbidity and mortality events in affected swine. The disease can take one of several forms (acute, chronic, or prenatal) and depending on the virulence of the inoculating strain may result in a lethal infection irrespective of the form acquired. Because of the disease-free status of the United States and the high cost of a viral incursion, a summary of US vulnerabilities for viral introduction and persistence is provided. The legal importation of live animals as well as animal products, byproducts, and animal feed serve as a potential route of viral introduction. Current import regulations are described as are mitigation strategies that are commonly utilized to prevent pathogens, including CSFV, from entering the US. The illegal movement of suids and their products as well as an event of bioterrorism are both feasible routes of viral introduction but are difficult to restrict or regulate. Ultimately, recommendations are made for data that would be useful in the event of a viral incursion. Population and density mapping for feral swine across the United States would be valuable in the event of a viral introduction or spillover; density data could further contribute to understanding the risk of infection in domestic swine. Additionally, ecological and behavioral studies, including those that evaluate the effects of anthropogenic food sources that support feral swine densities far above the carrying capacity would provide invaluable insight to our understanding of how human interventions affect feral swine populations. Further analyses to determine the sampling strategies necessary to detect low levels of antibody prevalence in feral swine would also be valuable.

## Key Points

- Classical swine fever (CSF) is currently a foreign animal disease in the United States and the economic consequences associated with an introduction could be severe.
- The virus is endemic in many parts of the world, including Central America, Africa, Asia, and parts of South America.
- Classical swine fever virus is most likely to be introduced to the US *via* the legal or illegal importation of animals or their products.
- Feral swine involvement would significantly challenge disease control and eradication methods.
- Effective live attenuated vaccines are available for use; however, antibodies generated against the current vaccines cannot be differentiated from those generated during a natural infection which complicates control and eradication methods.
- Future research should involve (1) density mapping of feral swine populations in the US as well as long-term studies on a fine spatial scale to evaluate contact dynamics, and movement ecology based on habitat and seasonality, of feral swine as these components are important for contact and subsequently, disease transmission, (2) studies to evaluate the effect of anthropogenic food sources on home range and density of feral swine, and (3) expanded analyses to explore sampling strategies needed to detect low levels of CSF antibody prevalence.

## Introduction

Classical swine fever (CSF), historically called hog cholera, is caused by CSF virus (CSFV) and can result in high morbidity and mortality in swine. This disease is reportable to the World Organization for Animal Health (OIE) and viral detection can severely diminish pork exports. The United States is currently free of CSFV, with the last reported case in 1978 (1). This manuscript outlines what is known about CSFV and aims to describe existing gaps in knowledge. Additionally, a summary of vulnerable sites of CSFV introduction into the United States and persistence within is provided.

### Virus Description

Classical swine fever virus is a small, enveloped RNA virus that belongs to the *Flaviviridae* family and as such, is closely related to bovine viral diarrhea virus (BVDV) in cattle and border disease virus (BDV) in sheep (2). The genome contains 12,300 base pairs and comprises four structural and seven non-structural proteins (3, 4).

### Transmission and Clinical Disease

Both domestic swine (and their feral counterparts) and wild suids, including javelina, bush pigs, and warthogs are susceptible to CSF. Natural and experimental infections have shown that suids are also capable of transmitting the virus (5, 6). Transmission routes include oronasal transmission through direct or indirect contact with infected pigs, the consumption of feed contaminated with virus, or *via* vertical transmission from infected sow to her offspring (7, 8). The virus is shed from all mucosal surfaces making sexual transmission a possibility. Pork and other pig products are a reservoir for CSFV and survival may be prolonged in heavily proteinaceous environments, especially that of cooled or frozen meat products (9–11). The infection can cause a range of clinical signs from an inapparent, subclinical infection to a hemorrhagic fever-like illness with high mortality (6). The incubation period is typically 7–10 days following infection; however, under field settings it is likely that a herd infection may not be detected for 2–4 weeks, primarily because of limited clinical signs and infrequent monitoring (8).

Classical swine fever strain differences have been observed and attempts have been made to categorize strains as highly virulent (those that kill nearly all pigs irrespective of other factors), moderately virulent (those that cause a sub-acute illness in postnatally infected piglets and sometimes cause abnormalities in fetuses), or avirulent (those that are attenuated and apathogenic in fetuses) (12). However, this classification system is incongruent with other findings where the degree of pathogenicity varies from one pig to another and is believed to be a response to host age (and immune status), viral strain, and inoculating dose (13, 14).

Very little is known about molecular or antigenic properties of the virus that are involved in determining virulence despite numerous sequencing and phylogenetic studies; however, characteristics have been described *in vitro* that allow for some viral virulence determination (12, 15). Virulent strains grow optimally at 39–40°C, moderately virulent strains grow optimally at 35–38°C, and low virulent strains grow optimally at 33–34°C. Highly virulent strains have also been found to grow faster and to higher titers compared to the other CSFV strains in cell culture and are more resistant to heat treatment (12). Furthermore, viral virulence can be artificially abrogated using laboratory techniques and specific proteins and post-translational modifications have been found to play an important role in viral virulence. The recoding of the structural glycoprotein E2 using codon usage deoptimization has been found to result in complete virus attenuation and is capable of protecting against a virulent CSFV challenge (16). p7 is a non-structural, hydrophobic polypeptide that, through the use of reverse genetics, has been found to be pore-forming and is involved in viral virulence (17). Finally, the three glycoproteins E^RNS^, E1, and E2 were evaluated for the effects of post-translational modifications and those that were not glycosylated failed to induce a detectable virus neutralizing antibody response and did not protect against virulent CSFV (18). Despite our capacity to make targeted mutations that result in complete viral attenuation, the exact properties that contribute to viral virulence remain unknown.

Infection with CSFV typically takes one of three forms: acute, chronic, or prenatal (8) and age, clinical signs, and disease outcome are listed in Table 1. Piglets, less than 12 weeks of age, often develop an acute infection characterized by fever, anorexia, lethargy, conjunctivitis, respiratory signs, and constipation followed by diarrhea as well as neurological signs that often include a staggering gait, hind end paresis, ataxia, and convulsions. Death follows 1–3 weeks after the onset of clinical disease (19). With increasing age the clinical signs of an acute infection are less specific and recovery is possible (8). The chronic form develops when pigs are unable to develop an effective immune response. The initial signs are similar to those observed in the acute phase, but as the infection persists the clinical signs become nonspecific, often including intermittent fever, chronic enteritis, and wasting. Pigs may survive 2–3 months before succumbing to the infection and shed virus consistently from viral incursion to death. The pre-natal form occurs when the virus crosses the placenta and infects the fetus during any stage of pregnancy. Abortion, stillbirths, mummification, and malformations are common when the virus crosses the placenta during early pregnancy; however, if infected 50–70 days into gestation the piglets may become persistently infected. They often appear clinically normal at birth and may survive for several months (called late-onset CSF) prior to showing poor growth, wasting, and/or congenital tremors. These piglets are believed to be the most important cause of viral perpetuation within a population as they constantly shed large amounts of virus (20). This persistently infected phenotype can also be generated by an early postnatal infection with either a lowly or moderately virulent strain of CSFV. While the chronic and prenatal forms of CSFV are always lethal infections, acute infections with CSFV are not always lethal and outcome is dependent upon a myriad of factors, including host age and immune status, and virulence of the acquired strain, among other factors (21). The age component seems to be an important factor that heavily impacts disease outcome, with the same virus and dose potentially resulting in a nearly asymptomatic infection in adult or breeding animals but may cause nearly 70% mortality in young animals (Volker Moennig, Personal communication, 2016). To date, neither beneficial nor detrimental host reaction patterns have been defined, suggesting that the outcome is largely dependent on the immune response of the host, with age as a strong factor. Additionally, differences in pig breed have been evaluated relative to infection with CSFV and it was not found to be a strong predictor of disease course; further suggesting that individual differences are the main driver for the clinical course of infection (6).

**Table 1:** Description of each disease form of CSFV.

Age	Virulence of strain	Infection form	Clinical signs	Disease outcome	Reference
<12 weeks	High	Acute	Fever, anorexia, lethargy, conjunctivitis, respiratory signs, constipation followed by diarrhea, and neurological signs	Typically death	(8, 19)
>12 weeks	High to moderate		Less specific and less severe signs when compared with those in younger animals	Recovery is possible	

Any age	Low	Chronic	Similar to those in the acute phase but as infection persists, signs become non-specific and include intermittent fever, chronic enteritis, and wasting	Typically death	(8)

Neonatal piglets	Moderate to low	Prenatal (early gestation)	Abortion, stillbirth, fetal mummification, and malformations	Death	(8, 22)
Newborn piglets		Prenatal (days 50–70 gestation)	Normal at birth then begin to show poor growth, wasting, and/or congenital tremors		

Experimental infections using highly virulent, moderately virulent, and lowly virulent strains, classified as described above by van Oirschot (12), demonstrated that the quantity of highly virulent virus shed is far greater when compared with either moderately or lowly virulent strains and is shed from an earlier point of infection (23). Interestingly, a difference is not only observable in the timing and quantity of virus excreted but also in the type of excretions that contain virus. Highly virulent strains are shed *via* all secretions and excretions while lowly virulent strains are restricted to oronasal secretion routes. This variation is thought to be due to viral tropism. Highly virulent strains spread rapidly throughout the body whereas lowly virulent strains are restricted to specific target organs. Mittelholzer et al. (15) developed a clinical score scheme for CSFV infections in pigs that allows for the quantification of observable clinical signs which includes 10 signs that are ranked between 0 (normal) and 3 (severe clinical sign) with a maximal score of 30. Using this clinical scoring format in conjunction with pyrexia it was found that highly virulent strains have clinical signs >15 and a fever ≥41°C, moderately virulent strains have clinical signs between 5 and 15 and a fever between 40 and 41°C, and lowly virulent strains have clinical signs below 2 and a fever ≤40°C.

While limited data is available for infection of wild boar with CSFV, it is widely assumed that there are no substantial differences between domestic pigs and wild boar in terms of susceptibility and clinical manifestations (21) and the reports that exist concur with this assertion. An experimental inoculation using Eurasian wild boar of various ages and sexes found that the acute course of the disease was independent on the origin of the isolate and that clinical signs varied strongly, both of which have been found in domestic swine (24). Chronically infected suids, those which shed copious volumes of virus for 2–3 months prior to succumbing to infection, serve as a reservoir in domestic swine; however, it is unknown if chronically affected wild boar could survive in their environment, and as such, how much of a role they may play in transmission of CSFV (21). Furthermore, pregnant wild boar sows infected during gestation were found to yield persistently infected piglets (25); although, the role congenitally infected piglets play in CSFV transmission in wild populations is likely limited due to their short survival time (26).

### Geographical Distribution

Classical swine fever is endemic in many parts of the world in both domestic swine and wild boar. As a reportable disease, information on specific countries and their annual CSF case load can be found at the OIE website (27). Canada and the United States are disease free and have been for 50+ and 30+ years, respectively (28). Mexico is recently disease free; however, Central America (excluding Panama and Belize which are disease free) is endemically infected, with control maintained through vaccination. Much of South America is endemically infected; however, countries are implementing control strategies such as vaccination, laboratory testing, stamping out, quarantine, control of transit, and import regulations which appear to be facilitating progress toward disease eradication. CSFV is present in Cuba, Haiti, and the Dominican Republic and control practices have been tried and, to date, have failed due in large part to a lack of funding and institutional support. Excluding Japan, CSF outbreaks occur with frequency in Asia and Southeast Asia, with the largest viral diversity found in these regions (1). Africa is believed to be CSF free; however, Madagascar has historically reported cases. Western Europe, specifically European Union member states, have sought progressive eradication throughout the twentieth century and vaccination was banned in 1990; however, the region is not CSFV free due to endemic infection in wild boar, especially in the Baltic states (Latvia and Lithuania), which is transmitted to domestic pigs through direct or indirect contact or swill feeding (1, 28, 29). In Eastern Europe, CSF remains a problem and vaccination in conjunction with stamping out is used to curb outbreak events (1, 28).

In 1997, there was an outbreak of CSFV in the Netherlands which resulted in direct economic losses of $2.3 billion and the death of approximately 9 million pigs (30). The virus is believed to have entered in mid-late December 1996, although the first case of CSFV was not detected until the middle of January and was not confirmed by laboratory diagnosis until the beginning of February. The primary case was at a mixed sow and finishing herd with nearly 1,500 pigs of varying ages in a very pig dense region of the country. A contaminated transport lorry from Germany is believed to have initiated the outbreak but the disease quickly spread between farms in the Netherlands and was exported to Italy, Spain, and Belgium. Routes of transmission that were believed to play an important role in the outbreak were the purchase of infected animals, transport vehicles, personnel, rendering plant cadaver collection service, artificial insemination (contaminated semen), pig slurry, neighborhood transmission, and other unknown factors; the disease was re-eradicated in March 1998. It has since been shown that neighborhood transmission (transmission between herds located within several kilometers of one another) presents a tremendous problem and modeling tools can be used to determine the risk of local transmission patterns (31).

### Immune Response to CSFV

Classical swine fever virus targets endothelial cells, lymphoreticular cells, macrophages, and some types of epithelial cells (2). Severe leukopenia is a characteristic finding associated with the early stages of CSFV infection, especially affecting lymphocytes (32). Reduced numbers of circulating B cells (33) and CD4^+^ and CD8^+^ T cells (34) have been observed prior to the onset of viremia and the function of T cells isolated during a CSFV infection were found to have compromised function, which is believed to be driven by apoptotic events (35–37). *In vitro* experiments have demonstrated that CSFV readily replicates within endothelial cells where it promotes a strong pro-inflammatory and pro-coagulatory response (38). If a similar process occurs *in vivo* it is suspected that the host immune response plays an important role in the hemorrhagic pathogenesis of the disease. Granulocytopenia has also been observed within several days of infection with CSFV in both peripheral and bone marrow-derived neutrophils which is thought to be a result of hematopoietic cell death likely due to indirect virus-host mediated mechanisms (39). Microarray analysis following infection with CSFV in swine macrophages and found 79 genes that had altered patterns of expression within 48 h of infection (40). Most of the expression patterns that were changed were found to be involved in the development of the innate immune response.

In young pigs there is a strong correlation between serum IFN-α and the acute disease process, which also directly correlates to the degree of lymphopenia; thus suggesting that high levels of IFN-α do not control the virus but, in fact, may mediate immunopathology (37). The release of pro-inflammatory and vasoactive mediators by macrophages following infection is an important contributor for CSFV pathogenesis. Dendritic cells are likely to release pro-inflammatory cytokines as well as large quantities of IFN-α and IL-12 which promotes T_H_1 activation.

## Domestic Swine in the US

**Figure 1:**
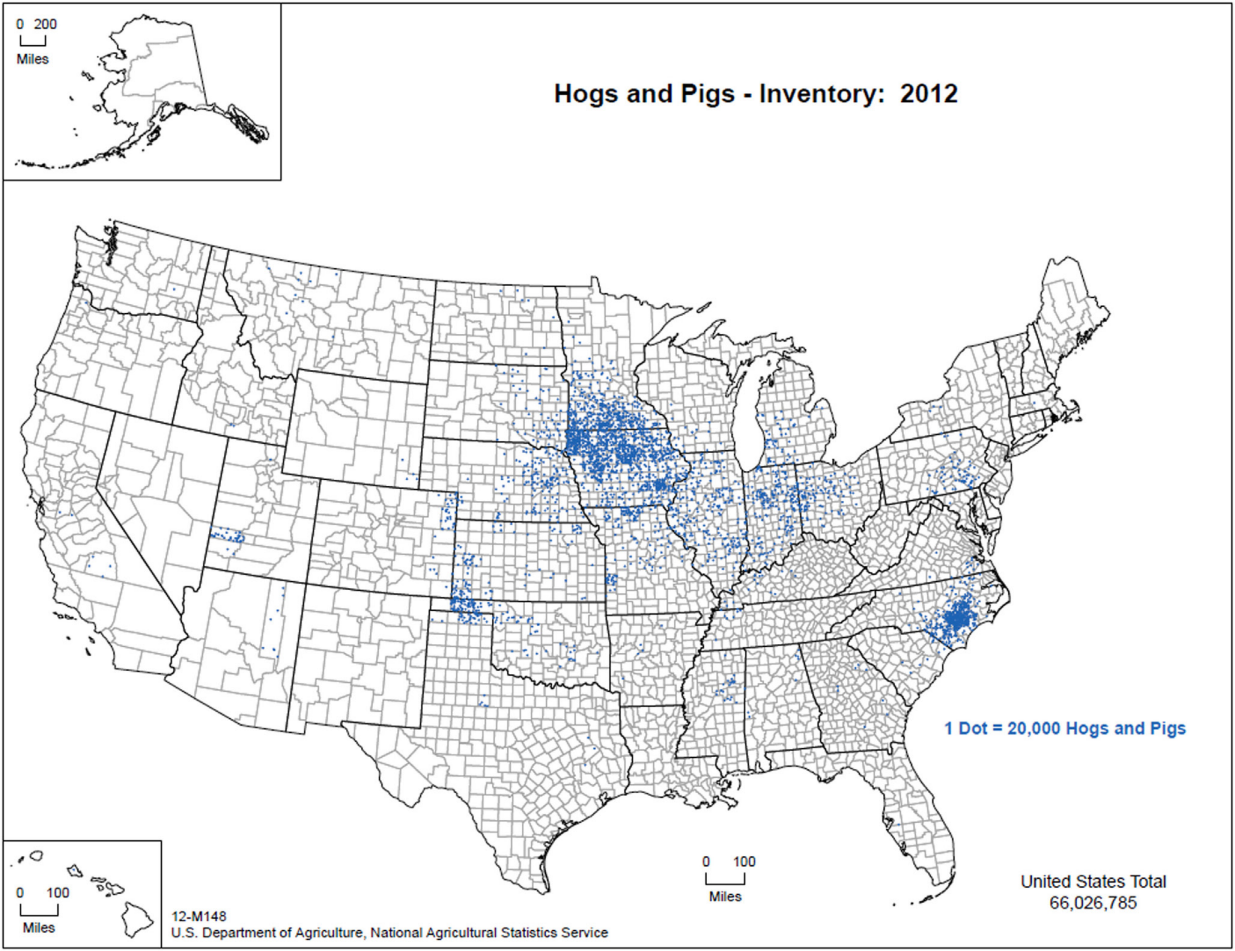
Distribution of pig production within the US, 2012 (figure courtesy of: United States Department of Agriculture, National Agricultural Statistics Service (44)—used with permission).

The commercial production of swine involves high biosecurity in a vertically integrated industry from farrowing through slaughter (National Pork Producers Council, Personal communication, 2016) A majority of the 65 million pigs in the United States are managed indoors under these conditions with Iowa, North Carolina, Minnesota, Illinois, and Indiana boasting the top pork production annually [Figure 1; (41)]. Strict rules exist relative to the management of animal feed, transport vehicles, personnel, and other fomites as a means of preventing cross-contamination. Despite regimented biosecurity practices, porcine epidemic diarrhea virus entered the United States in 2013 and has since been traced back to contaminated feed bags, suggesting that the commercial swine industry may not be as refractory to pathogens as previously thought (42). This viral introduction and subsequent spread, suggest that viral stability plays a crucial role in the effectiveness of the biosecurity practices and highly stable pathogens may not be adequately safeguarded against (43).

Hobbyists and backyard farmers are likely to have domestic swine that are not managed under intensive conditions and may be exposed to environmental elements and a number of other species (45). Furthermore, it is possible that their feed may be more diverse and likely involves less oversight when compared with commercial operations. The map of domestic pig production in the US depicts high densities of domestic swine and indicates where a CSF introduction would likely be most problematic to the swine industry (Figure 1).

## Eurasian Wild Boar and CSFV

Native to much of Europe and Asia, Eurasian wild boar pose a challenge for the control and eradication of CSFV in that region. Experimental inoculations have demonstrated that they are acutely susceptible to the virus but their exact role in maintenance or transmission events to domestic swine remains unknown (6). Large scale culling events of domestic swine due to CSF occurred in Austria, Belgium, the Czech Republic, Germany, Italy, Spain, and the Netherlands between 1991 and 2001. It has been suggested that CSF in wild boar is associated with the persistence of the disease in domestic swine, although viral persistence in either population can readily be transmitted to the other (46). Interestingly, serum samples collected from 259 wild boar in Croatia in 2003 found that 47% of the pigs sampled were positive for the presence of CSFV antibodies using a highly specific ELISA (47). There was a statistically significant difference in CSFV seropositivity recorded between age groups with pigs less than 1 year having the highest likelihood of being seropositive, which may be reflective of the presence of maternal antibodies. These data suggest that the virus is either being maintained within the population and infects the young after maternal antibodies wane or that the virus is being constantly reintroduced from domestic swine infected with CSFV ((47); Volker Moennig, Personal communication, 2016).

In France, wild boar were sampled between 1992 and 2002 as part of a compulsory monitoring project to track CSF infection in the native suid fauna (48). Originally viroprevalence was used to estimate incidence; however, low incidence makes finding a viremic animal very rare as virus can only be detected for a few weeks following infection. Conversely, antibodies can be detected for extended time periods following infection and recovery. Antibody prevalence was monitored over the ten year period with overall seroprevalence used to estimate the proportion of immune wild boar, seroprevalence in juveniles used to estimate the incidence, and seroprevalence in different age classes used to estimate incidence evolution in a given cohort. Spatial and temporal trends found that after 2000, no seropositive juveniles were detected and that the epizootic was regionally extinct. Using seroprevalence in juveniles to estimate incidence is likely an underestimation of the true incidence as CSFV is acutely virulent in juveniles such that most would die prior to antibody development; however, it is believed to be a useful metric given the limitations associated with sampling wild species and the short duration of a viremia. A capture-mark-recapture study confirmed the above findings, such that most (80%) wild boar piglets that become infected with CSFV succumb to infection within two weeks and those that survive infection (20%) recover quickly (49). Host innate immunological factors were found to be associated with fitness in wild boar, with high complement activity associated with the highest probability of survival (49). No chronic CSFV infection was observed in wild boar piglets (49).

Historically, the viral kinetics of CSFV entering a naive wild boar population appear to proceed to extinction within a few years of viral incursion; however, recent outbreaks observed in France, Germany, and Italy were characterized by a mortality peak succeeding the initial infection followed by a slow progressive decrease in the infection rate over a long time period (46). Laddomada (50) articulated that there are two main factors that contribute to either an epizootic event or a persistent, endemic infection: (1) the availability of susceptible animals (which is influenced by population size, herd immunity, and age structure and dynamics) and (2) the frequency of infectious contacts (which is influenced by density and animal movements). Artois et al. (46) articulate that wild boar are unlikely to serve as a true reservoir species because, (1) eradication of CSFV from domestic populations typically results in disease disappearance from wild suids, (2) intentional release of CSFV was performed in wild, free ranging boars and was not found to persist, and (3) when appropriate epidemiological data was collected regarding the outbreak among wild boars, human errors (feeding, burying of contaminated carcasses, among others) were found to be involved in nearly every case. However, wild boar density is important and factors into the role that these native suids play in viral maintenance and transmission. In high density regions the disease tends to become endemic, whereas in lowly dense regions it often dies out over time. Young wild boar whose maternal antibodies have waned are believed to be responsible for the majority of transmission as older animals are already immune, either as a result of vaccination or having survived a natural infection. Hunting targeted at reducing the young wild boar population can be used to diminish the number of susceptible hosts which can also be useful in curbing an outbreak event (Volker Moennig, Personal communication, 2016).

Feeding of wild boar in Europe has risen in popularity which results in both more interaction among the wild boar as well as population sizes that may exceed the natural carrying capacity. Factors particular to a specific epidemiological scenario play an important role in the capacity for wild boar to become endemically infected: population density, frequency of interaction, and social structure, among others (48). Furthermore, frequent re-introductions from infected domestic swine may give an impression that the disease has become endemic within the wild boar population (Volker Moennig, Personal communication, 2016).

The control of disease in wildlife is often very challenging, however, vaccines can be used to combat infectious disease by decreasing the proportion of susceptible animals below a threshold necessary for disease maintenance within the population (49). The C-strain live attenuated vaccine (discussed in detail below) has been found to be highly efficacious and palatable baits have been developed for oral delivery in wild boar. In order to curb an outbreak of CSFV in wild boar in Germany in 2009, a vaccine regimen was developed which involved three double campaigns in spring, summer, and autumn (51). The protocol was designed to maximize both antibody titers and the proportion of vaccinated juvenile wild boar, as such, an initial bait was dropped followed by a booster 28 days later. The C-strain vaccine is derived from a genotype 1 strain whereas the circulating field virus was a genotype 2 strain; thus, using a multiplex real-time RT-PCR assay with partial sequencing assay vaccinated animals could be differentiated from those naturally infected. This strategy depends on the epidemiological setting as regions with genotype 1 viruses circulating would not be able to use this multiplex assay to differentiate.

Developing an oral bait that is detectable (odor, color), palatable (odor, taste), and that is effectively ingested are all crucial components for a successful mass oral vaccination program and quite difficult (49). Despite their omnivorous diet, wild boar were found to prefer baits containing plant derived compounds when compared with animal derived compounds. To further complicate this program, it is necessary that the vaccine be released in the oral cavity such that the tonsils can initiate the immune response. The vaccine must be perforated by pig teeth prior to swallowing; bait size is crucial as too small will likely be swallowed prior to perforation and too big will limit the number of animals that uptake the vaccine. Field trials were first performed in Germany in the 1990s and were then deployed to other European countries during the 2000s. Prebaiting, the practice of accustoming wild boar to the bait prior to vaccine distribution, was found to be necessary and the vaccine bait was delivered by hunters to account for wild boar foraging which occurs in groups, such that concentrating baits in feeding places was far more effective when compared with random distribution by aircraft.

## Diagnostics

The diagnosis of CSF typically consists of four complementary elements (although all four are not always detectable) which include field clinical signs, gross pathology findings, indirect detection (serology), and direct detection (virus isolation or antigen or nucleic acid detection) (52). Live virus, as well as RNA, can be detected from blood, tonsil swabs, or tissues upon necropsy (19). Samples collected from live animals should be taken when the animal is pyrexic as it substantially increases the probability of viral detection. The OIE manual recognizes a myriad of assays as acceptable means for detecting CSFV, such as a fluorescent antibody test, immuno-peroxidase staining, antigen capture ELISA, virus isolation in cell culture, and RT-PCR; some assays are designed to evaluate live virus or viral particles while others detect CSFV-specific antibodies (19, 53–55).

Antibodies are first detectable 2–3 weeks following initial infection and often persist for the duration of the life of the pig (52). Serological assays are highly useful for both diagnostics and surveillance and the OIE recommends a fluorescent antibody neutralization test, a neutralizing peroxidase linked assay, or an antibody ELISA (55). Viral neutralization assays are regarded as the “gold standard” but they are labor intensive and require cell culture capabilities, which are not always available. ELISAs for CSFV diagnosis are typically designed for the E2 glycoprotein and this assay type is heavily used as a screening tool for antibodies during and after outbreaks, monitoring CSFV infection in wild boar populations, and to evaluate vaccine coverage following an oral administration in wild pigs (52). Despite widespread use, the majority of well-established traditional assays used to detect CSF virus or antibodies are not validated by OIE standards as the first chapter outlining methods of validation for diagnostic assays was not published until 1996 (55). It is important to note that antibodies for both BVDV and BVD can cross react with CSFV-specific antibodies and in some cases, serological assays may provide an inaccurate read unless specifically guarded against (56).

## Vaccines

Currently, multiple live attenuated vaccines are commercially available and have been found to be safe and highly efficacious against infection with CSFV (57). As an example, the lapinized Chinese vaccine (C-strain) was developed in the 1950s by Chinese researchers and has been used extensively to control CSF in China and many other countries (58). Despite providing sterilizing immunity to nearly all vaccinees within one week of vaccine administration, current live attenuated vaccines for CSF have a major disadvantage: it is impossible to differentiate between naturally infected and vaccinated animals using serological methods (55, 59). This is concerning as vaccines are being used to supplement other control methods and viral transmission cannot be effectively modeled due to the lack of assays capable of differentiating between vaccinated and infected animals. However, Zhao et al. (58) describe a multiplex real-time RT-PCR assay that is both rapid and sensitive for differentiating between wild-type viruses and the C-strain vaccine for CSFV in China. This assay is only applicable for C-strain based vaccines, and is not capable of distinguishing between other exotic vaccines; with further work, it remains a possibility that differentiating assays may become available for more live attenuated CSFV vaccines that work on a global scale.

As a means of circumventing the primary concern associated with live attenuated vaccines, several other vaccine strategies have been employed, such as the generation of immunogenic CSFV particles, DNA vaccines, viral vectors expressing CSFV proteins, chimeric pestiviruses, and trans-complemented deleted CSFV genomes (57). These novel vaccines require a myriad of doses and dosages to attain various levels of protection against CSFV challenge. E2 has been found to be a highly antigenic envelope glycoprotein that can be expressed using a baculovirus expression system and has been found to induce a neutralizing antibody response in pigs (60, 61). Importantly, animals vaccinated with the recombinant E2 vaccine, also referred to as a subunit vaccine, can be readily differentiated from those naturally infected as the latter will generate a polyclonal response that involves E2 in addition to NS3 and E^RNS^ whereas vaccinated animals will develop a monoclonal response against E2 exclusively (62). An ELISA has been developed that detects E^RNS^ and can effectively be used to differentiate samples from vaccinated and infected animals (55, 63). Two E2 vaccines are currently licensed by the European Agency for the Evaluation of Medicinal Products and commercially available despite the higher risk of persistently infected animals and the need for increased caution with pregnant sows (63). In addition to the subunit E2 vaccines, chimeric pestivirus vaccines have been evaluated and promising results were found for CP7_E2 alf (which is a BVDV backbone expressing the CSFV E2 glycoprotein) and flc11 (a CSFV backbone with the E^RNS^ gene replaced by the corresponding BVDV gene) (64). Both of the aforementioned pestivirus candidates were comparable to the C-strain vaccine and upon early challenge, CP7_E2 alf was found to be better for safety and efficacy following oral administration. The marker concept has been demonstrated but the discriminatory assays require further optimization.

Field efficacy of the CP7_E2 alf vaccine was evaluated using experimental infections of both Eurasian wild boar and domestic swine (65). Following vaccination, experimental domestic swine or wild boar are typically challenged with a CSFV genotype 1.1 strain, which represents a homologous virus to the vaccine strain but is unlikely to be reflective of circulating field isolates. Vaccination with CP7_E2 alf followed by challenge with virulent CSFV genotypes 2.1 and 2.3 led to complete protection which affirms the field applicability of the chimeric pestivirus vaccine. Furthermore, longevity of immunity studies were undertaken to evaluate the duration of protection following vaccination with CP7_E2 alf followed by infection with a virulent CSFV strain (66). Domestic swine were vaccinated orally or *via* intramuscular injection with CP7_E2 alf and challenged with a virulent CSFV strain six months following vaccination. Antibody titers were stable for the duration of the 6 months irrespective of the route of vaccination and high antibody titers lead to full virus neutralization and full protection following lethal challenge was observed. One non-responder was observed following oral vaccination, suggesting that the oral route of vaccination leads to a more variable response. The CP7_E2 alf vaccine is a very promising prophylactic and its capacity to be differentiated from a natural infection provides a tremendous advantage. This vaccine is currently licensed by the European Medicines Agency.

## CSF and the United States

The domestic swine industry in the United States would likely be very negatively impacted in the event of CSFV introduction. Passive and active surveillance programs, defined as using reports and testing of animals found dead and developing a program to capture animals in some way (live-capture, hunter harvest, euthanasia) for testing, respectively (67), exist for both domestic and feral swine. USDA Veterinary Services have outlined a surveillance program for domestic swine and the objectives are as follows: (1) surveillance for rapid detection of CSFV in US swine, (2) monitor the risk of introduction of CSF into US swine, (3) surveillance of international CSF status, and (4) surveillance to document freedom of CSF (68). Passive surveillance is performed in all states and requires involvement by producers, diagnosticians, and slaughterhouse inspectors, among others to report and sample any suspect cases. Unthrifty pigs, considered to be those that gain weight poorly or are otherwise somewhat sickly, are often sold to off market vendors and APHIS field staff or other cooperating personnel collect tonsil samples in these markets as a way to survey for infectious agents, including CSFV. This method is deemed to be an effective surveillance strategy as pigs from surrounding regions are often consolidated in these markets which makes for an efficient means of sampling sickly pigs from a wider geographical area. Furthermore, high risk areas, which include regions with garbage feeding operations, backyard swine operations, feral swine hunting clubs, military bases, international air or sea ports, farming operations utilizing an international labor force, and/or corporations engaging in international swine movement, are subject to active surveillance protocols; 25 states are considered high-risk. All garbage feeder operations in the United States are licensed and regularly inspected and heat treatment of all feed is mandatory. Texas and Florida are considered particularly high risk and as such, two swine slaughter establishments in Florida and three in Texas randomly collect blood which is sent to the Foreign Animal Disease Diagnostics Laboratory for further testing, especially from pigs in the southern portion of each state, light weight pigs, or those in transition.

Feral swine are also surveyed as a preventative and early sentinel in the event of a CSFV intrusion. Feral animals are typically referred to those who have been phenotypically selected by humans but do not live under human supervision or control (69). Feral swine (*Sus scrofa*) describe a genotypically diverse composite of suids that include escaped domestic swine, truly wild Eurasian boars, and their hybrids (70, 71). There are ongoing surveillance programs for CSFV antibodies in feral swine and targeted areas typically include domestic hog production areas and landfills. Counties are weighted based on the presence or absence of each of the aforementioned criteria (72). This type of targeted surveillance increases the probability of early detection in the event of virus introduction (73, 74). Samples are collected *via* culling operations as well as from hunter-killed pigs and serology is performed to evaluate the presence of CSFV antibodies. Serological assays are used in series, beginning with an ELISA, followed by an immunoperoxidase test, and finally virus neutralization. As soon as a sample tests negative, it is no longer assayed (e.g., if a sample is negative at the ELISA it is not tested by immunoperoxidase or virus neutralization). This series of diagnostic assays exists as antibodies against BVDV and BDV cross react with CSFV which may result in a false-positive reading; thus, the downstream diagnostics are increasingly specific.

In addition to domestic and feral swine surveillance efforts, plans have been developed in the event of viral incursion (75). The primary document outlines the four key outbreak strategies: (1) stamping out, which involves depopulating clinically affected animals and in-contact susceptible animals; (2) stamping out with emergency vaccination to kill, in which clinically affected and in-contact susceptible animals are depopulated and at-risk animals are vaccinated and subsequently depopulated and disposed of; (3) stamping out with emergency vaccination to slaughter, in which clinically affected and in-contact susceptible animals are depopulated and at-risk animals are vaccinated and slaughtered and processed; or (4) stamping out with emergency vaccination to live, which involves depopulating clinically affected animals and in-contact susceptible animals and vaccinating at-risk animals whence they are not depopulated. Multiple factors influence the response strategy, including scale of the outbreak, outbreak consequences, acceptance, available veterinary countermeasures, and available resources for implementing response strategies. A detailed approach is also needed on how to provide relevant information to responders and stakeholders during an outbreak (76).

The United States also harbors a supply of CSFV vaccines at the National Veterinary Stockpile. The US maintains both the C-strain live attenuated vaccine as well as the CP7_E2 alf vaccine (Personal communication, 2016). The C-strain vaccine has been used extensively in control and eradication programs in many countries throughout the world and its efficacy, safety, onset of immunity, duration of immunity, and many other factors are well characterized in the field. The CP7_E2 alf vaccine has been used to a much lesser extent in field applications and performance is less well characterized. Each product requires one vaccination to result in sterilizing immunity; however, as discussed above in the vaccine section, antibodies generated in response to vaccination with the C-strain vaccine cannot be differentiated from those developed by animals who are naturally infected whereas the CP7_E2 alf vaccine allows for differentiation.

## Summary of Vulnerabilities for the Introduction or Persistence of CSFV in the US

### Risk of Introduction into the United States

**Figure 2:**
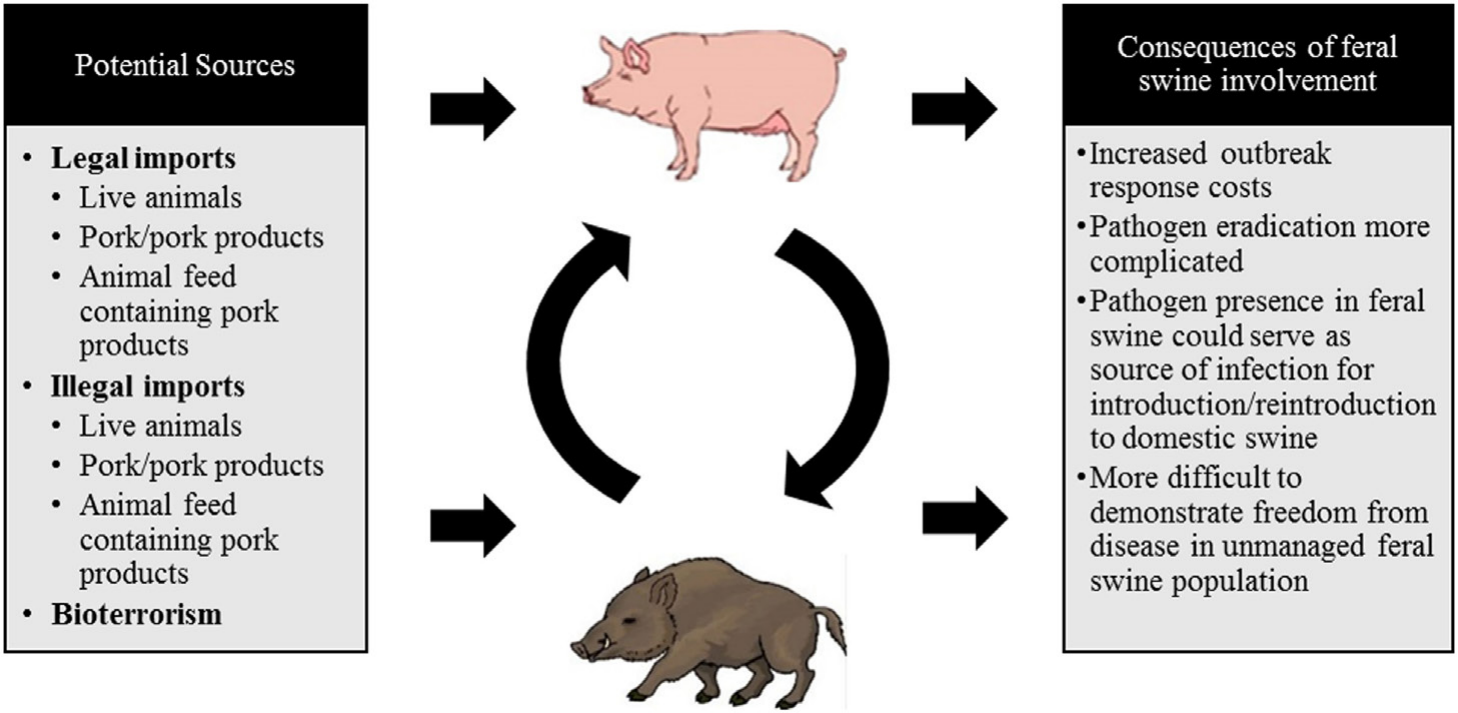
A schematic diagram depicting the potential sources of a classical swine fever virus (CSFV) incursion into the US and the consequences of feral swine involvement.

The legal movement of live animals or their products, byproducts, or animal feed, the illegal movement of live animals and their products, or an intentional viral release in an act of bioterrorism are all channels through which a disease outbreak of CSFV is likely to occur. These routes are the most probable means of introduction due to patterns of virus transmission, viral stability, and current global instability. Each of these possible routes of introduction are displayed in Figure 2 and described in detail in this section.

#### Legal Movement of Live Animals

In 2015, the only live swine imports into the United States (domestic or exotic/wild species) came from Canada in which just under 6 million pigs were imported for breeding, feeding, or direct to slaughter. Canada is CSFV free so it is unlikely that a live pig imported from the northern border would be responsible for a viral incursion.

#### Legal Movement of Animal Products, Byproducts, and Animal Feed

A number of animal products, byproducts, and feed are imported into the United States annually and permits are required. Treatments typically involving heat, pH, or fixation processes are required for all animal products and byproducts that are being imported from CSF-endemic regions. Unprocessed products are permitted for entry from CSF-free countries. Specific temperature and duration cooking is required for animal feed imported from countries endemic for CSF. The European Union is designated as a low-risk region and as such, products can be imported raw; although documentation is required to certify that the product is coming from an unaffected herd in an unaffected region.

#### Illegal Movement of Live Animals and Their Products

The risk of virus introduction through illegal transport of live animals and their products is related to the types of products being moved, and their country of origin and final destination. The US Department of Homeland Security Customs and Border Protection (CBP) confiscates products and specimens from domestic animals in the cargo and express courier environments. More than 68,000 products and specimens were confiscated by CBP between 2012 and 2016 and over 90% of confiscations were of products originating in Asia and Europe. These findings confirm that illegally imported products and specimens serve as a route for CSFV introduction as Asia and parts of Europe are endemic for the virus.

The confiscation of illegally imported wildlife and their products fall under the jurisdiction of the US Fish and Wildlife Service. Between 2006 and 2016, 133 wild suid products were confiscated and the majority of the products seized were from Africa. It is important to note that a tiny fraction of all illegally imported wildlife shipments are believed to be detected (77); thus, these numbers likely represent a gross underestimation.

#### Bioterrorism

Classical swine fever virus is a bioterrorism candidate because of the tremendous economic value of the domestic swine industry in the United States, the clinical disease associated with infection, the endemic status of many countries globally making viral acquisition a ready option, the robust stability of the virus in a proteinaceous environment and the crippling implications for international trade. As such, domestic and feral swine must be surveyed for disease frequently and systems for rapid diagnosis must be readily available.

### Factors That Complicate Eradication Efforts following Introduction

Feral swine are found in large numbers across much of the US and their highly flexible diet, interaction with domestic pigs, and unmanaged lifestyle make them an opportune vector for further disease transmission following an introduction event. In the event of a viral incursion feral swine could contribute to amplification and transmission events to other feral swine or their domestic counterparts and would serve to significantly complicate disease control measures (78).

#### Feral Swine

Feral swine include released domestic swine, truly wild Eurasian boar, and their hybrids (79). Nearly 6 million feral swine roam the US and are believed to be found in at least 35 states. They have been shown to carry a wide variety of pathogens capable of infecting domestic livestock (80) and GPS data has shown feral swine interacting with domestic swine (45). These types of interactions increase the risk of pathogen transmission from feral to domestic swine. Alternatively, the evaluation of interspecies interactions using GPS and proximity loggers between cattle, domestic pigs, Eurasian wild boar, and red deer found very limited interactions between wildlife and livestock (81), although feral swine may not behave as Eurasian wild boar relative to their social behavior. Peccaries are not included as a risk as they are not believed to be important for virus maintenance and transmission (82).

While domestic swine are likely the higher risk group for acquiring CSF, it is important to note that feral swine could participate in a CSFV outbreak in the US in one of the several ways. Feral swine regularly are found scavenging in landfills and consumption of CSFV-contaminated garbage (e.g., airport waste) could lead to an introduction event directly into feral swine. The index feral swine could then infect other feral swine as well as domestic pigs, particularly those housed in a backyard setting. Alternatively, some domestic pigs are fed swill and illegally imported or improperly treated swill could result in a CSFV introduction directly into domestic swine which could then spillover into feral swine.

As described previously, feral swine are routinely culled and samples are collected as part of an active surveillance program to ensure rapid detection and diagnosis in the event of a CSFV incursion. Swafford et al. (83) published antibody data against CSFV in feral swine and found that no antibodies were detected in collection years 2007 and 2008. In fact, no CSFV-specific antibodies have been detected in feral swine since the inception of the program through the current day (Kerri Pedersen, Personal communication, 2016). In the event of a CSFV introduction, large sample sizes are required to detect low prevalence pathogens and sampling on a country-wide scale, with a feral swine population exceeding 6 million animals, would necessitate an extremely large sample size and subsequently, a substantial and sustained economic investment. Simulation models conducted in Germany demonstrated that the financial resources and personnel necessary for reliable testing are substantial and difficult to sustain over time. In addition, sufficient sample sizes to detect low virus prevalence are difficult to obtain (84). Large and costly efforts would be needed to test a statistically significant portion of the population, which would require significant funding and a continuous effort over time.

Disease-emergence dynamics modeled in feral swine, a highly gregarious and social species (85), demonstrated that under realistic demographics and contact structure, a CSFV-like disease could persist for long periods of time resulting in many more cases (78). These data are in agreement with a model that demonstrated that CSFV in wild pigs in Australia was able to both persist and spread across the landscape (86). These findings support the assertion that feral swine present a concern for viral incursion because of their behavior—omnivorous and opportunistic diets, close social interaction, and unmanaged movement—and that despite active surveillance efforts, infection may remain silent until a substantial portion of the population displayed antibody presence or morbidity or mortality events. However, introduction of CSFV into a naive population is often accompanied by high morbidity and mortality as observed by the detection of an increase in the number of dead animals on the landscape, which would presumably be detected and investigated by field biologists (87).

Disease transmission modeling in feral swine must account for their social activities and lifestyles. Sounders are comprised of reproductively active females and their young while males typically live a solitary life. GPS data has shown that contact rates are much higher for animals within the same sounder when compared with those animals in different sounders (88). Sounder interaction is reduced at distances >2 km and disease transmission is also expected to be reduced (89). These findings suggest that the quarantine surrounding a positive premise should be at least 2 km. Importantly, lone boars tend to have much larger home ranges (90) which could complicate quarantine and surveillance efforts in the event of a disease outbreak. It is important to note that under the assumption that transmission of CSFV among wild boar occurs primarily through direct contact, CSF incidence should increase with increasing host density and should go extinct under a threshold density of susceptible hosts. Interestingly, however, analysis of the incidence and viral persistence of CSF in the French Vosges Forest demonstrated that infection depressed density but did not support the hypothesis of density dependence of incidence (91). This suggests that the presence of circulating CSFV reduced the population of wild boar but that viral transmission was not strictly density dependent. However, these findings may not be representative of all ecological settings as density has appeared to be a crucial factor for CSFV transmission in a multitude of other studies (2, 8, 21).

Feral swine are of tremendous concern in the event of disease outbreaks that affect domestic livestock. Hog panels have been evaluated and can effectively contain feral swine (92). Although relatively cheap and quick to erect, fencing is only a viable option to control movement of feral swine on a small scale.

Targeted culling of young wild boar would likely not be efficacious in the United States to curb an outbreak as the entire US feral swine population is susceptible to infection with CSFV when compared with the scenario in Western Europe where older animals are typically immune as a result of vaccination or a survived infection with CSF.

In many instances in the United States, vaccination of susceptible animals following the introduction of a foreign animal disease is not a viable option for a myriad of reasons; however, it could be an option in the event of a widespread outbreak. Vaccination using oral baits would be a potential strategy to curb an outbreak in feral swine as they have been used very effectively in Eurasian wild boar in the European Union.

**Figure 3:**
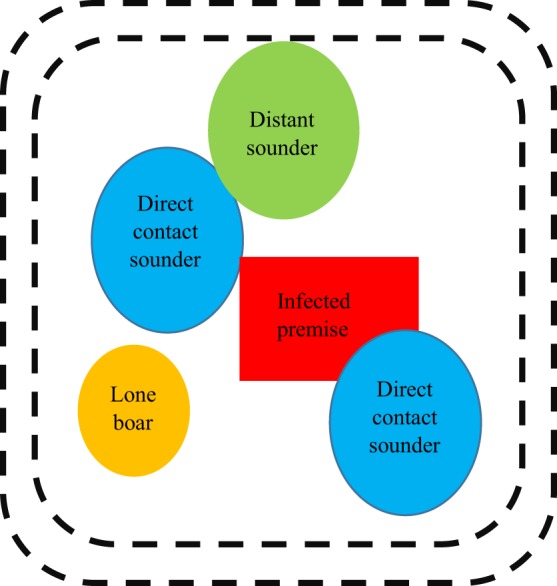
Example illustration of how disease management may be approached in a feral population. It is important to note that this graphic is not drawn to scale (e.g. sounders contain more pigs but have smaller ranges when compared with lone boars) and that all of the groups depicted in this graphic are dynamic and in constant movement.

Outbreak specific characteristics would be important to include, such as the amount of time that has elapsed since the first case, the virulence of the CSFV strain, the density of both domestic and feral swine, among many other components. Multiple control and mitigation strategies could be employed in the event of a foreign animal disease introduction that may have spilled over into feral swine populations (Figure 3).

Sounders that are in direct contact with swine from the infected premise (“direct contact sounders”) are included as well as “distant sounders” which are likely to interact with “direct sounders.” A lone boar is also included in this depiction and often have a much larger home range when compared with sounders; they are likely to be interacting with domestic swine on the infected premise, “direct contact sounders,” and “distant sounders,” as well as sounders that exist outside of this graphic and other boars. The exact distance of the perimeter fencing (demarked as black dotted lines) would be determined based on factors related to sounder home range, the environment, and pathogen specific components; however, the below diagram provides insight into how disease management may be approached in a feral population. A double perimeter fence would be employed as CSFV can be readily transmitted by direct contact; thus, it would be essential to prevent feral swine within the quarantine region from interacting with feral swine outside the quarantine region. The graphic has been simplified to include only one infected premise within the quarantine area; however, this is unlikely to be accurate in rural regions of the United States where hobby farms are abundant.

Disease surveillance in feral and wild animals is challenging for a number of reasons and control and eradication measures are abundantly complicated. Resource allocation and a systemic perspective is useful when making important decisions in the absence of information (93). Currently both the northern and southern borders in the United States are porous, which in the event of a CSFV introduction into either Canada or Mexico, the fluidity of feral swine moving across the border region will be challenging.

## Concluding Remarks

Due to the severe morbidity and mortality caused by CSFV in domestic swine, introduction (either accidental or purposeful) presents a risk to the United States. Federal oversight is provided for the importation of live animals and their products and comprehensive mitigation strategies are mandated for products originating in CSFV-endemic countries. Despite these safeguards, the illegal importation of animals and their products is an avenue that is inherently unrestricted; thus, serves as a viable route for viral introduction. An act of bioterrorism is also a potential route of viral introduction. Active surveillance of domestic swine and efficient channels for disease reporting are imperative to allow for the rapid diagnosis of infectious disease in swine.

Classical swine fever virus introduction in feral swine would complicate management and eradication. Unrestricted movement of feral swine along the US, Canada, and Mexico borders could be an issue in the event of a virus introduction into any of the three countries. Simulation models have demonstrated that CSFV-like pathogens are likely to persist and spread across the landscape in feral swine populations (78, 86) and despite active antibody surveillance it is likely infeasible to sample the appropriate number of feral pigs in order to reliably determine that CSFV is not present in the population (84); although the sampling is likely sufficient for a morbidity or mortality event. However, developing detailed population and density maps for the feral swine population in the United States would be useful in the event of a viral incursion. This knowledge could be harnessed to anticipate risk of transmission to domestic livestock or wildlife species and to determine strategies for preventing spread and eradicating the virus in the specific region of concern. In conjunction with population density mapping, long-term studies on a fine spatial scale should be conducted to evaluate contact dynamics, movement ecology based on habitat, and seasonality of feral swine. Kramer-Schadt et al. (94) articulate that knowledge of social structure, dispersal, and population densities are key to understanding epidemics. Furthermore, studies evaluating anthropogenic causes of clustering in feral swine (e.g. landfills, bait stations, etc.) would be very useful in determining the distance of attraction for these food sources and how their presence alters both the natural carrying capacity for feral swine as well as their behavior and any related changes in home range. Finally, further analyses should be performed to determine the sampling protocols necessary to detect low levels of CSF antibody prevalence in feral swine.

## Author Contributions

VB was involved in the development of the idea, collection of the data, data analysis and interpretation, and preparation of the document. SB was involved in idea development and document preparation.

## Conflict of Interest Statement

The authors declare that the research was conducted in the absence of any commercial or financial relationships that could be construed as a potential conflict of interest.
